# *Trypanosoma rangeli*: a new perspective for studying the modulation of immune reactions of *Rhodnius prolixus*

**DOI:** 10.1186/1756-3305-2-33

**Published:** 2009-07-17

**Authors:** Eloi S Garcia, Daniele P Castro, Marcela B Figueiredo, Fernando A Genta, Patrícia Azambuja

**Affiliations:** 1 Laboratório de Bioquímica e Fisiologia de Insetos, Instituto Oswaldo Cruz, Fundação Oswaldo Cruz, Avenida Brasil 4365, Rio de Janeiro, 21045-900, RJ, Brazil

## Abstract

Insects are exposed to a wide range of microorganisms (bacteria, fungi, parasites and viruses) and have interconnected powerful immune reactions. Although insects lack an acquired immune system they have well-developed innate immune defences that allow a general and rapid response to infectious agents.

Over the last few decades we have observed a dramatic increase in the knowledge of insect innate immunity, which relies on both humoral and cellular responses. However, innate reactions to natural insect pathogens and insect-transmitted pathogens, such as parasites, still remain poorly understood.

In this review, we briefly introduce the general immune system of insects and highlight our current knowledge of these reactions focusing on the interactions of *Trypanosoma rangeli *with *Rhodnius prolixus*, an important model for innate immunity investigation.

## Introduction

### The insect innate immune reactions

There are two types of innate immune reactions: (i) the humoral response that is related to antimicrobial peptides, lectins and the prophenoloxidase (PPO) cascade and (ii) the cellular response which includes phagocytosis, hemocytes aggregation and encapsulation of pathogens.

Innate immunity of insects relies on a limited variety of receptors which recognize specific compounds that are on the surface of microorganisms or are released by them. The most well known pathogen-associated molecular patterns (PAMPs) are microbial cell-wall components like lipopolysaccharides (LPS) of Gram-negative bacteria, lipoteichoic acid and peptidoglycans of Gram-positive bacteria, β-1,3 glucans from fungi as well as glycosylphosphatidylinositol (GPI) from protozoan parasites [1,2].

The humoral immune system recognizes PAMPs by pattern recognition receptors which are conserved in evolution to bind unique products of microbial metabolism not produced by the host [1,2]. The humoral pattern recognition receptors such as LPS-binding proteins, peptidoglycan recognition proteins (PGRPs), Gram-negative binding proteins (GNBPs), β1,3-glucans recognition protein (βGRP), circulates in the hemolymph of insects [3,4].

In the hemocyte surface there are several proteins implicated in the cellular immune response against invading microbes by recognizing the PAMPs. The most well known cellular receptors involved in recognition of pathogens in several insect species are croquemort (homologue of the mammalian CD36 family), Down syndrome cell-adhesion molecule (Dscam), peptidoglycan recognition protein (PGRP-LC), Eater (transmembrane protein) and the Toll family members [3,4].

### Humoral immunity

*Drosophila melanogaster*, a dipteran, has become an appropriate model for the investigation of immune pathways and insect-microorganism interactions [4-6]. Apparently, the main components of the core signaling processes are conserved between insects [4]. The genome sequencing of these insects allowed a comparative genomic analysis of the gene families involved in the *Drosophila *defence reactions [7]. The best-characterized insect humoral response is the production of antimicrobial peptides (AMPs). These peptides are small, cationic and with different structures. They are released into the hemolymph during infection [8]. The main source of AMPs is from the fat body, but several epithelia and insect organs are also able to produce these substances [9]. The most important AMPs are defensins which act mainly against Gram-positive bacteria [10]. However, cecropins that have a large spectrum are more effective against Gram-negative bacteria [11]. There are other AMPs like attacin, diptericin, drosocin and drosomycin, etc [5,12]. Most AMPs have simple and non-specific modes of antibiotic action, such as driving pathogen membrane disruption by altering the membrane permeabilization or through an intracellular target [10-12].

**Figure 1:**
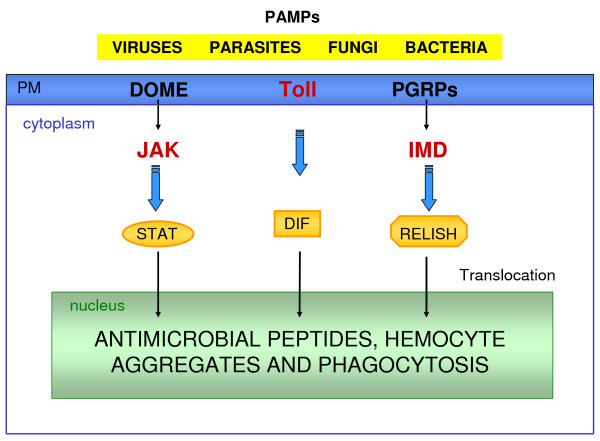
**Toll, IMD and JAK-STAT pathways**. Insect tissues recognize pathogen-associated molecular patterns (PAMPs) by transmembrane receptors (DOME, Toll and PGRPs) in plasmatic membrane (PM) that activate the three pathways. The JAK-STAT pathway is activated by the receptor DOME (*domeless*) that transduces the signal to JAK and the cytosolic STAT. The Toll pathway starts with activation of the receptor Toll that signals to the cleavage of Dorsal-related immunity factor (DIF) complex releasing DIF. The IMD pathway through peptidoglycan recognition proteins (PGRPs) activates IMD (immune deficiency) that regulates the proteolytic cleavage and activation of Relish. The transcription factors (STAT, DIF and Relish) translocate to the nucleus through the nuclear membrine activating the expression of its transcriptional targets resulting in the production of antimicrobial peptides and other immune responses.

Investigation in *Drosophila *demonstrated that production of AMPs is related to two distinct pathways: Toll and IMD pathways [3]. Recent studies suggested that these two pathways respond respectively to Gram-positive or Gram-negative bacteria and fungal infections in insects [5,12]. A third pathway involved in immune reactions, especially in mammals, is the JAK/STAT (Janus kinase/Signal transducer and activator of transcription) [13]. The JAK/STAT signaling pathway takes place mainly in the fat body of insects. The production of AMPs is a common result of JAK/STAT, Toll and Imd pathway activity [14] (Figure 1).

**Figure 2:**
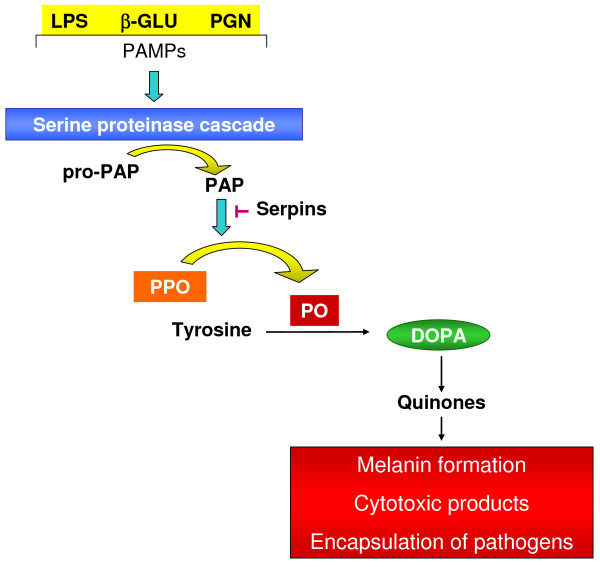
**A serine proteinase cascade is activated when different receptors recognize pathogen-associated molecular patterns (PAMPs)**. These serine proteases hydrolyze and activate the prophenoloxidase-activating proteinase precursor (proPAP) to prophenoloxidase-activating proteinase (PAP) that can be inhibited by serpins (proteinase inhibitors). The enzyme PAP hydrolyses prophenoloxidase (PPO) releasing phenoloxidase (PO). PO oxidizes tyrosine to dihydroxyphenylalanine (DOPA) and subsequently into quinones, the precursors of melanin, cytotoxic products and encapsulation of pathogens.

The prophenoloxidase (PPO) cascade, which leads to melanization and production of highly reactive and toxic compounds (e.g. quinones), is another important humoral immune reaction in insects. Also, there are several papers reporting that phenoloxidase (PO) promotes cellular defence reaction like phagocytosis [for review see [15]]. Although in some cases, the melanization process is not important for clearing an infection, it is relevant for pathogen encapsulation [15]. Melanization depends on tyrosine metabolism. The PPO activation cascade is composed of several proteins, including PPO, serine proteases and their zymogens, as well as proteinase inhibitors. The PPO cascade is set off by the recognition of PAMPs that leads to the activation of a serine protease cascade culminating in the limited proteolytic cleavage of PPO to produce active PO that catalyzes the oxidation of tyrosine to dihydroxyphenylalanine (DOPA) which is subsequently oxidized to form dopaquinone and dopamine quinone as well as 5, 6-dihydroxyindole which have highly antibacterial activities (Figure 2). These compounds are precursors of the melanin polymer which is deposited on the surface of encapsulated parasites, hemocyte nodules and wound sites [13]. Besides the PPO activation cascade is regulated by plasma serine protease inhibitors (including members of the serpin superfamily) and active phenoloxidase (PO), this process being directly inhibited by proteinaceous factors [15,16] (Figure 2). Such regulations are essential because the products of PO activity are potentially toxic to the host.

Finally, the mosquito *Anopheles stephensi*, a natural vector of human malaria, limits parasite development with inducible synthesis of nitric oxide (NO). Elevated expression of *A. stephensi *NO synthase (NOS) that is highly homologous to other characterized NOS genes, occurs in the midgut and carcass soon after invasion of the midgut by *Plasmodium *[17]. Interestingly, in the hemolymph the nitrite/nitrate ratios, and the products of NO synthesis are higher in *Plasmodium*-infected mosquitoes and the treatment with NOS inhibitor N-nitro-L-arginine methylester significantly increases the number of parasites in infected mosquitoes [17].

### Cellular reactions

Insect cellular responses are mediated by circulating hemocytes, and they include phagocytosis, hemocytes aggregation and encapsulation. Insect phagocytosis refers to the process by which hemocytes recognize, internalize and destroy microorganismal invaders [18]. In *Drosophila*, phagocytosis is performed mainly by plasmatocytes, while hemocyte aggregation and encapsulation are carried out by lamellocytes that attach, embrace and inactivate the invading organisms, which then die by asphyxiation or by free radical attack [19]. Frequently, there is a local activation of the PPO cascade that cross-links the hemocyte aggregates and microorganisms in a melanin envelope.

### Eicosanoid pathways

Eicosanoids are oxygenated metabolites of arachidonic acid with a huge range of physiological functions in a diversity of organisms. Among the important functions ascribed to eicosanoids are the central role that they play in the inflammatory and immune defence reactions in mammals [20] and the mediation of cellular defence responses to bacterial infections in insects [21]. In fact, results from over 20 insect species representing 5 orders indicated that eicosanoids mediate cellular immune reactions to bacterial infections [22]. Stanley-Samuelson *et al*. [23] demonstrated, for the first time, that eicosanoids regulate bacterial clearance from the insect's hemolymph. Following this pioneering paper, much research has been done on the relation of eicosanoids in regulating the insect immune system, especially on the elimination of inoculated bacteria from the hemolymph by nodule formation, the major cellular immune response to bacterial infections in insects [21-24]. The decrease of arachidonic acid production due to dexamethasone effect on phospholipase A_2 _(PLA_2_) activity reflects on the products of the cyclooxygenase (COX) and lipoxygenase (LOX) pathways, diminishing both bacteria clearance [23] and nodulation [24] in insects. After that, the recognition of the biological significance of eicosanoids in signal transduction in insect immune responses rapidly increased [21] with studies of insects infected with bacteria and fungi [25-27], parasitoids [28], protozoa [29,30] and viruses [31,32].

**Figure 3:**
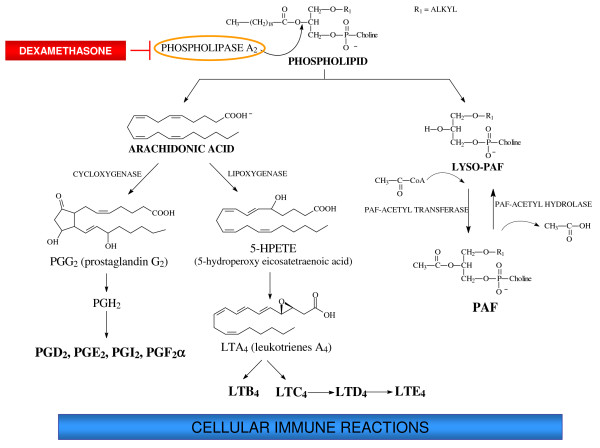
**Phospholipids are hydrolyzed by phospholipase A_2 _liberating arachidonic acid and Lyso-PAF, regulators of insect's immune system**. Arachidonic acid is the substrate for eicosanoid production, prostaglandins via cyclooxygenase and leukotrienes via lipoxygenase. Lyso-PAF is acetylated by PAF-acetyl transferase releasing PAF that can be degraded by PAF-acetyl hydrolase that hydrolyses PAF regenerating Lyso-PAF. In the presence of dexamethasone the immune responses are inhibited due to the suppression of phospholipase A_2 _activity with lower production of eicosanoids and PAF. On the other hand when exogenous arachidonic acid is added there is enhancement of eicosanoid production and immune responses increase.

Miller and Stanley [33] have shown that eicosanoid biosynthesis inhibitors have a direct effect on *Manduca sexta *hemocytes and Tunaz *et al*. [34] demonstrated that dexamethasone exerts its effect on insects by inhibiting PLA_2_. Investigations made by Mandato *et al*. [25] showed that eicosanoid biosynthesis inhibitors attenuated the PO activity in *Galleria mellonella *challenged with bacteria, and this inhibitory effect of dexamethasone was abolished by the addition of arachidonic acid (Figure 3). So, many models of insect species have been studied to expand and generalize the hypothesis that eicosanoids mediate the nodule formation in insect hemolymph during immune responses to bacterial, fungal, parasitoid and viral infections.

Some invading microorganisms induce an immunological depression to avoid the immune reactions of insects. The entomopathogenic bacterium, *Xenorhabdus nematophila*, induces immunodepression in target insects by (PLA_2_) activity inhibition causing lethal septicemia of the infected hosts [35-37].

## The *Trypanosoma rangeli *model

Trypanosomes are digenetic parasites that have insects as vectors and infect human beings and other vertebrates as hosts [38]. So far, only species of the genus *Rhodnius *have presented infective forms of *Trypanosoma rangeli *in their salivary glands [39]. In Latin America, this parasite has two major lineages based on kinetoplast DNA (kDNA) markers: one group presents three types of kDNA minicircles (KP1, KP2 and KP3- *T. rangeli *KP1+), while the other group has only KP2 and KP3 minicircles (*T. rangeli *KP1-) [for review [40]]. *T. rangeli *is a harmless parasite for humans and various wild and domestic animals, but it can be pathogenic to the insect vector [40].

**Figure 4:**
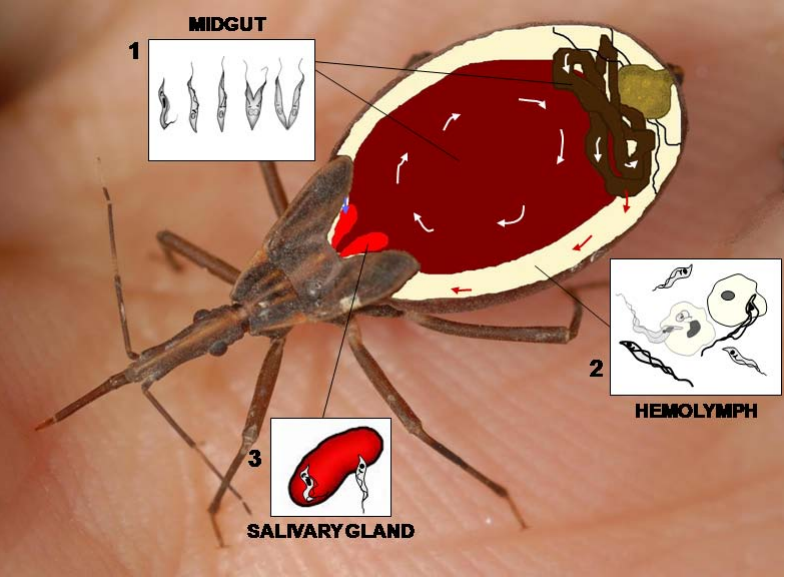
**Scheme of biological cycle of *Trypanosoma rangeli *within its insect vector**. The insect feeds on blood infected with trypomastigote forms which differentiate to epimastigotes in the midgut (white arrows) where they multiply (1). Some epimastigotes invade the hemolymph through the gut epithelium (red arrow). Long and short forms of epimastigotes can entry into the hemocytes and multiply or replicate in the plasma (2). Some parasites invade the salivary glands (blue arrow) and differentiate to trypomastigotes which will be transmitted when the insect-vector feeds on another host (3).

While the full biological cycle of *Trypanosoma cruzi*, the causative agent of Chagas disease, takes place in the guts of the triatomine vectors, and the infecting parasites are eliminated with feces and urine to contaminate vertebrate hosts [41-44], the *T. rangeli *life cycle in the vector is different. The vector infection begins when parasitesare ingested as trypomastigote forms. The parasites multiply as epimastigotes in the gut, and they are able to penetrate through the gut epithelium [45] (Figure 4) invading the hemocele. In the regular course of infection, a few days after parasite ingestion, short epimastigote forms appear in the insect hemolymph. Soon, they disappear to be replaced by a massive colonization by long epimastigotes [46]. The epimastigotes survive in the hemolymph and/or inside the hemocytes. Then, they migrate and possibly by recognition of carbohydrate moieties attach to salivary glands [47], invade them and complete their development into infective forms [48-50] (Figure 4).

Interestingly, Garcia *et al*. [50] described that *T. rangeli *impairs *R. prolixus *salivary gland function, preventing full expression of its antihemostatic machinery [51] prolonging the duration of intradermal probing.

## The *Rhodnius prolixus *model

*Rhodnius *belongs to the subfamily Triatominae of the family Reduviidae that is made up of 140 species of triatomines, several of which are vectors or potential vectors of the hemoflagellate protozoan parasites *T. cruzi *and *T. rangeli*. However, only species of the genus *Rhodnius *have infective forms of *T. rangeli *in their salivary glands [48,52].

Since the main characteristic of the *T. rangeli *life cycle is the invasion of the insect hemocele it must overcome the immune reactions of its vector. We will therefore use the bloodsucking bug, *Rhodnius prolixus*, as a tool for providing insights into how insects defend themselves against infection by bacteria and parasites such as *T. rangeli*. From a practical point of view, *R. prolixus *has many advantages as an insect model for research on parasite transmission. These include simple maintenance and rearing in the laboratory and feeding through an artificial membrane device. This facilitates the infection with parasites and, due the body size, they are easily handled and manipulated [42,53]. Besides that, *R. prolixus *is frequently used for physiological studies [54,55] and, more recently, for biochemical and immunological investigations [44]. Nevertheless, there is a great lack of molecular data about the *R. prolixus *immune system, and the majority of studies focused in the cellular response or in the effects of the vector immune defences in the parasite development (and vice versa). However, the sequencing of its complete genome (670 MBp) [56] will facilitate the application of advanced molecular biology to enhance ongoing research for exploration of biomedical significance of this insect.

## *Trypanosoma rangeli *infection and *Rhodnius prolixus *immune reactions

Knowledge on the *Rhodnius *immune system and its activation in response to microorganism infections has grown in recent years. The first defences against microbiological infections are the structural barriers outside or inside the body (for example, exoskeleton and the perimicrovillar membrane in the midgut [55,57-59].

The establishment of *T. rangeli *infection in both gut and hemocele of the insect vector is possibly regulated by a range of biochemical and physiological processes. The first environment for the transformation and development of *T. rangeli *is in the gut. There the parasites are confronted with anterior and posterior midgut components and products of blood digestion. These included bacteria [60,61], hemolytic factors [62] and lectins [46,63], all of which may modulate the infection of *T. rangeli *in the vector gut.

Once in the hemocele, *T. rangeli *must overcome the robust insect vector's defence system including lysozymes and trypanolytic activities [46], PPO activation [64], phagocytosis and hemocyte microaggregate formations [29,30,65-67], agglutination [46,63], superoxide and nitric oxide production [68] and a trypanolytic protein which acts specifically against the *T. rangeli *KP1-strains [40]. All these activities seem to act as biological barriers raising difficulties for the development and transmission of the parasite in the vector.

### Humoral reactions and T. rangeli infection

Although Lopez *et al*. [69] showed that defensin was induced both in the hemolymph and midgut of *R. prolixus *by inoculation of *Escherichia coli *and *Microccocus luteus*, there are some data on the inactivation of the *R. prolixus *humoral immune system by parasite infection. Mello *et al*. [46] demonstrated that, after systemic inoculation of *T. rangeli *short epimastigotes into the hemocele of *R. prolixus*, the parasite produces a high intensity of infection through successive division during the extracellular development, with a concomitant increased levels in the lysozyme activity in the hemolymph. They also showed that *T. rangeli *infection induced neither trypanolytic nor peptide antibacterial activities, but a galactose-binding lectin from *R. prolixus *hemolymph, which enhanced the activation of clump formation by *T. rangeli *in *R. prolixus *hemocyte monolayers. An increase in clump size and hemocyte aggregation was also described [70]. This purified lectin also affected *in vitro *the motility and survival of *T. rangeli *culture short forms, but not the long forms [70], which are predominant in the hemolymph two days after inoculation [46].

Another important biological event of *T. rangeli *interference in the insect immune reactions is its ability to activate the PPO system of *R. prolixus*. Gregorio and Ratcliffe [71,72] demonstrated that *Triatoma infestans*, but not *R. prolixus*, presents a very active PPO system when activated by laminarin and lipopolysaccharides. For both species of insects, neither *T. rangeli *from culture nor parasite lysates were able to trigger PPO activation *in vitro*. However, the presence of the parasite in *R. prolixus *hemolymph assays reduced the level of PPO activation by laminarin. These authors suggest that the susceptibility of *R. prolixus *to *T. rangeli *hemolymph infection may, at least in part, be explained by the suppression of the inset immune defence system i.e. inhibition of the PPO cascade in the presence of this parasite.

Interestingly, Gomes *et al*. [73] clearly demonstrated using *in vitro *experiments that the activation of the PPO pathway occurred when the hemolymph was incubated with fat body homogenates and short epimastigote forms of *T. rangeli*. The same authors using *in vivo *experiments showed that short, but not long, epimastigote forms activated directly the formation of melanin [73]. In addition, the PPO-activating pathway was suppressed when insects, which had been fed on blood containing either short or long epimastigotes, were challenged by thoracic inoculation of the short forms. This indicates that the reduction of the PO activity was a result of parasite ingestion. The PPO pathway is activated when glycosylphosphatidylinositol (GPI) anchors, specifically glycoinositolphospholipids (GIPLs) and GPI-mucins purified from *T. rangeli *epimastigotes, are inoculated in the insect [74].

One factor that can be important for killing *T. rangeli *is nitric oxide and nitrite/nitrate radicals, products of NO synthase (NOS) activity. Whitten *et al*. [68] described experiments to demonstrate whether or not nitric oxide and superoxide production could operate during *T. rangeli *infection in *R. prolixus*. These authors followed the inoculation of two strains and two developmental forms of *T. rangeli *after 24 h. When the H14 strain was inoculated, the parasites failed to multiply and invade the salivary glands whilst the Choachi strain rapidly multiplied in the hemolymph to invade salivary glands. However, in insects inoculated with H14 strain, the levels of PPO and superoxide generated by *R. prolixus *were significantly higher than Choachi strain, and nitrite and nitrate levels were also much higher with H14 inoculations. Usually, short forms of epimastigotes stimulated greater superoxide and PPO reactions than long epimastigotes in both parasite strains in the hemolymph of *R. prolixus*. Furthermore, when the NADPH oxidase inhibitor, N-ethylmaleimide, or the inhibitor of the inducible nitric oxide synthase, S-methylisothiourea sulfamide, are injected into *R. prolixus*, they resulted in higher insect mortality after *T. rangeli *infection of either strains compared with those untreated controls [68]. Whitten *et al*. [75] demonstrated that the most pronounced reactions to crude LPS occurred in the *R. prolixus *fat body and hemocytes, while tissues of the digestive tract were most responsive to infections by *T. cruzi *and *T. rangeli*. This suggests that the NO-mediated immune responses in this insect are pathogen specific and independently modified both at the transcriptional and NO synthase gene expression.

It is interesting to note that in a screening of *R. prolixus *genes activated after *T. cruzi *infection by sequencing of subtractive libraries, no genes related to the humoral immune response were found to be transcriptionally upregulated [76]. These results suggest that the *R. prolixus *immune responses to parasites are not mediated by AMPs, and could be centered in hemocytes nodulation, encapsulation and phagocytosis. The comparison between the responses against bacteria and *T. cruzi *also showed that *R. prolixus *activates different mechanisms of defence depending on the pathogen [77]. In this way, it is possible that the regulation of immune related genes in *R. prolixus *differs significantly after *T. cruzi *or *T. rangeli *infection.

### Hemocyte microaggregation and phagocytosis and T. rangeli infection: role of eicosanoids and PAF pathways

The circulating hemocytes are essential for the insect immunity. In *R. prolixus *seven morphological hemocyte types were identified by phase-contrast microscopy: prohemocytes, granulocytes, plasmatocytes, cystocytes, oenocytes and adipohemocytes and giant cells [78]. Some cellular immune reactions have been studied in *T. rangeli*-triatomine interactions. Garcia *et al*. [29], demonstrated for the first time that eicosanoid biosynthesis inhibitors applied to *R. prolixus *strongly affect hemocyte microaggregation, one of the cellular immune reactions. The main data found by these authors were: (i) insects that had previously been fed on blood containing biosynthesis inhibitors of PLA_2 _(dexamethasone) and COX (indomethacin) and non-selective LOX inhibitor (nordihydroguaiaretic acid, NDGA) showed a significant increase in the number of free epimastigote forms of *T. rangeli *in the hemolymph and, consequently, increased lethality; and (ii) the parasite infection in insects treated with these compounds led to less hemocyte microaggregation and attenuated the activation of PPO system in the hemolymph.

Garcia *et al*. [29] suggest that arachidonic acid was not available in insects treated with dexamethasone. Indeed, the application of arachidonic acid significantly enhanced both hemocyte microaggregation and PO activity in the hemolymph of insects previously treated with dexamethasone and challenged with parasites. It also reduced the number of parasites in the circulation and the mortality of insects. The effects of indomethacin and NDGA were considered relevant because they indicated the influence of multiple eicosanoid metabolites in immune reactions of *R. prolixus *infected with *T. rangeli*. Furthermore, hemocelic inoculation of epimastigotes of *T. rangeli *into larvae of *R. prolixus *previously fed with blood containing the same parasite, demonstrated a reduced number of hemocyte microaggregates, enhanced the number of parasites in the hemolymph as well as increased the mortality of these insects. All these effects were counteracted by combined injection of *R. prolixus *with *T. rangeli *and arachidonic acid [30]. These results suggest that the arachidonic acid pathway can be a mediator of hemocyte microaggregation reactions in the hemolymph of insects inoculated with *T. rangeli *and that oral infection with this protozoan inhibits the release of arachidonic acid (Figure 3).

One interesting novelty of this parasite-vector interaction was revealed by Machado *et al*. [79]. They demonstrated that hemocelic injection of short *T. rangeli *epimastigotes in *R. prolixus *that were previously fed with blood containing WEB 2086 [a strong platelet-activating factor (2-acetyl-1-hexadecyl-sn-glycero-3-phosphocholine (PAF) antagonist] resulted in reduced hemocyte microaggregation, attenuated PPO activation in the hemolymph as well as increased the parasitemia and insect mortality. Nevertheless, simultaneous application of PAF did not counteract hemocytes microaggregation and PO activity.

It was demonstrated that physalin B, a natural secosteroidal chemical from *Physalis angulata*, induces immunodepression in *R. prolixus *[80-82] and strongly blocks hemocyte phagocytosis and microaggregate formations in *R. prolixus *[80]. The inhibition induced by physalin B was counteracted for both phagocytosis and microaggregation of hemocytes by arachidonic acid or PAF applied by hemocelic injection. Physalin B did not alter hemocyte PLA_2 _activities but it significantly enhanced PAF-acetyl hydrolase (PAF-AH) activity in the cell free hemolymph and hemocytes. Theses findings reinforce the importance of PAF and arachidonic acid pathways in cellular immune reactions in *R. prolixus *(Figure 3).

The most exciting outcome in the investigation of *T. rangeli *in triatomines is the PAF influence on the hemocyte nodulation [79] and phagocytic responses of *R. prolixus *hemocytes against *Saccharomyces cerevisiae *[66,67]. These authors evaluated the effects of PAF and eicosanoids in the phagocytosis in hemocyte monolayers (the main cell type implicated in this process is plasmatocytes) of *R. prolixus *against the yeast *S. cerevisiae*. The experiments demonstrated that the phagocytosis of yeast cells by *Rhodnius *hemocytes is very efficient in both controls and cells treated with PAF or arachidonic acid. However, phagocytosis of yeast particles is significantly diminished when the specific inhibitor of PLA_2_, dexamethasone, is applied to the hemocytes. By contrast, dexamethasone pre-treated hemocyte monolayers exhibit a drastic enhancement in the quantity of yeast cell-hemocyte internalizations when the cells are treated with arachidonic acid. Phagocytosis decreases expressively in hemocyte monolayers treated with WEB 2086, a specific PAF receptor antagonist. Nevertheless, a decrease of phagocytosis with WEB 2086 is also counteracted by the treatment with PAF [66,67]. The authors suggest that these data on phagocytosis of yeast cells by hemocytes are related to the activation of PAF receptors and provides a novel insight into the cell signaling pathway of non-self recognition related to cellular immune reactions in the insect-parasite relationship.

Finally, Figueiredo *et al*. [66] demonstrated that hemocyte phagocytosis was significantly reduced by oral infection with *T. rangeli*. These authors demonstrated that hemocyte phagocytosis inhibition caused by the parasite infection was rescued by exogenous arachidonic acid or PAF applied by hemocelic injection. They also observed an attenuation of PLA_2 _activities in *R. prolixus *hemocytes (cytosolic PLA_2_: cPLA_2_, secreted PLA_2_: sPLA_2 _and Ca^++^-independent PLA_2_: iPLA_2_) and an increase of sPLA_2 _in cell-free hemolymph. At the same time, the PAF-AH activity in the cell-free hemolymph enhanced considerably. These data suggest that *T. rangeli *infection depresses eicosanoids and insect PAF analogous (iPAF) pathways giving support to the role of PLA_2 _in the modulation of arachidonic acid and iPAF biosynthesis and of PAF-acetylhydrolase (PAF-AH) by reducing the concentration of iPAF in *R. prolixus *[67]. The relationship between the expression of the genes of PLA_2 _and PAF-AH as well as general cellular responses and signal transduction pathways is poorly understood in hemipterans. In this way, it is difficult to interpret the *T. rangeli *immunosuppression in terms of regulation of cellular signal transduction cascades. The data above suggest an inhibition of the NF-κB pathway, one well known effect of physalin treatment in mammal cells [82]. This is in agreement with the inhibition of the humoral immune response, but more detailed studies on the molecular mechanisms are needed to clarify this point.

All these finding illustrate the ability of *T. rangeli *to modulate the cellular immune responses of *R. prolixus *to favor its own multiplication in the hemolymph.

## Conclusion

**Figure 5:**
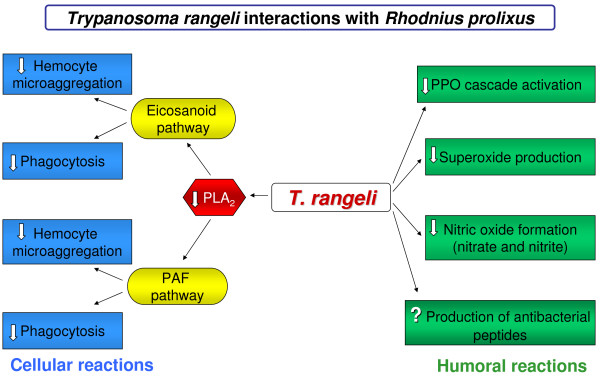
**A schematic illustration of *Trypanosoma rangeli *regulating the *Rhodnius prolixus *immune reactions**. White arrows (↓) indicate immune reactions decrease after infection of *R. prolixus *with *T. rangeli*. In the case of AMP production by *R. prolixus*, there is no known (?) regulation by *T. rangeli*.

Interventions to study the triatomine vector biology may be useful to develop new concepts and means to block parasite transmission, both of which are urgent and necessary. The recent investigations into *R. prolixus *immune reactions relating to *T. rangeli *development have established a new conceptual hypothesis: a fine modulation of insect factors can interfere with parasite development and this is important for the establishment of infection, being an attractive target for intervention (Fig. 5).

Despite the progress in understanding the complexity of the insect immune responses, our knowledge of this theme in hemipteran vectors remains far from complete. Much work is still needed to understand the successful transmission of protozoans as the result of the immune modulation, as caused by *T. rangeli *infection in *R. prolixus*. It is necessary to understand humoral and hemocytes-surface receptors and regulators and intracellular signaling molecules to permit the development of new immunomodulatory drugs, designed to control vector insect's populations.

Finally, another point to be considered is that for triatomines the limited use of molecular biology technology has permitted only a fragmented view of the immune defence system in this important Chagas disease vector. Moreover, advances in *Rhodnius *genomics and functional genomics in the near future will lead to a rapid development of this field. The study of genes involved in immune reactions will reinforce the need to better understand the defence responses related to parasite-vector interactions.

## Competing interests

The authors declare that they have no competing interests.

## Authors' contributions

All authors engaged in developing the manuscript and approved the final version.
